# Post-intervention acceptability of multicomponent intervention for management of hypertension in rural Bangladesh, Pakistan, and Sri Lanka- a qualitative study

**DOI:** 10.1371/journal.pone.0280455

**Published:** 2023-01-19

**Authors:** Tazeen H. Jafar, Saeideh Tavajoh, H. Asita de Silva, Aliya Naheed, Imtiaz Jehan, Chamini Kanatiwela de Silva, Nantu Chakma, Maryam Huda, Helena Legido-Quigley, on behalf of COBRA-BPS Study Group

**Affiliations:** 1 Program in Health Services & Systems Research, Duke-NUS Medical School, Singapore, Singapore; 2 Duke Global Health Institute, Duke University, Durham, NC, United States of America; 3 Clinical Trials Unit, Department of Pharmacology, Faculty of Medicine, University of Kelaniya, Kelaniya, Sri Lanka; 4 Initiative for Noncommunicable Diseases, International Centre for Diarrhoeal Disease Research, Dhaka, Bangladesh; 5 Department of Community Health Sciences, Aga Khan University, Karachi, Pakistan; Marie Stopes International, PAKISTAN

## Abstract

**Background:**

COBRA-BPS (Control of Blood Pressure and Risk Attenuation-Bangladesh, Pakistan, Sri Lanka), a multicomponent, community health-worker (CHW)-led hypertension management program, has been shown to be effective in rural communities in South Asia. This paper presents the acceptability of COBRA-BPS multicomponent intervention among the key stakeholders.

**Methods:**

We conducted post-implementation interviews of 87 stakeholder including 23 community health workers (CHWs), 19 physicians and 45 patients in 15 rural communities randomized to COBRA-BPS multicomponent intervention in in Bangladesh, Pakistan, and Sri Lanka. We used Theoretical Framework for Acceptability framework (TFA) with a focus on affective attitude, burden, ethicality, intervention coherence, opportunity cost, perceived effectiveness and self-efficacy.

**Results:**

COBRA-BPS multicomponent intervention was acceptable to most stakeholders. Despite some concerns about workload, most CHWs were enthusiastic and felt empowered. Physicians appreciated the training sessions and felt trusted by their patients. Patients were grateful to receive the intervention and valued it. However, patients in Pakistan and Bangladesh expressed the need for supplies of free medicines from the primary health facilities, while those in Sri Lanka were concerned about supplies’ irregularities. All stakeholders favoured scaling-up COBRA-BPS at a national level.

**Conclusions:**

COBRA-BPS multicomponent intervention is acceptable to the key stakeholders in Bangladesh, Pakistan and Sri Lanka. Community engagement for national scale-up of COBRA-BPS is likely to be successful in all three countries.

## Introduction

Hypertension is the leading attributable risk factor for mortality globally. Evidence suggests that lifestyle modification and pharmacological treatment can lower BP and reduce the risk of adverse cardiovascular disease outcomes (CVD) and kidney disease. However, BP control rates remain suboptimal globally [1, 2]. Less than 20% of those with hypertension have adequately controlled BP using conventional targets of <140/90 mm Hg [3].The problem is particularly concerning in low- and middle- income countries (LMICs) like Bangladesh, India, Pakistan, Myanmar, Nepal, and Sri Lanka as people of South Asians origin have been shown to have enhanced susceptibility to vascular disease [1, 4]. Moreover, two-thirds of South Asia’s population live in rural areas where health literacy is lower, healthcare infrastructure is weaker, and CVD case fatality rates are significantly higher compared to urban areas [5].

Recently, we reported findings from COBRA-BPS, a cluster randomized controlled trial in 30 rural communities in three South Asian countries (Bangladesh, Pakistan and Sri Lanka) over 2 years and demonstrated that a community health worker (CHW) led multicomponent intervention was more effective in lowering systolic BP by 5 mm Hg versus usual care in adults with hypertension [6]. The projected per-capita cost of multicomponent intervention is less than $2 per-capita annually to scale-up making it an affordable and potentially sustainable intervention [6].

The pre-implementation interviews of stakeholders identified a number of barriers in accessing hypertension care in the 3 countries, which have also been reported from many other LMICs. Some of the key barriers include lack of awareness about hypertension, poor patient and healthcare provider communication between patient and healthcare provider, poor access to quality care and anti-hypertensive medications, long travel time to clinics, long queues at the clinics, and inadequate training of healthcare providers and insufficient resources for management of hypertension [7–11].

Although the COBRA-BPS multicomponent intervention was designed to address barriers to hypertension management and was effective and cost-effective in lowering BP, it remains unclear if the multicomponent intervention was also acceptable to the stakeholders after their experience in delivering and receiving the intervention.

We aimed to explore the post-intervention acceptability of COBRA-multicomponent intervention from key stakeholders’ perspectives, including healthcare providers (community health workers and physicians) and patients from three countries.

We used the Theoretical Framework of Acceptability (TFA) which focuses on affective attitude, burden, ethicality, intervention coherence, opportunity cost, perceived effectiveness, and self-efficacy [13]. This information, coupled with cost-effectiveness, is critical for scaling up COBRA-BPS intervention in the three countries.

### Materials and methods

#### Conceptual framework

Acceptability is a multi-faceted construct [12]. It reflects the degree to which an intervention is considered to be appropriate by the intervention deliverers (physicians, community health workers (CHWs) called Health Assistants in Bangladesh, Lady Health Workers in Pakistan, and Public Health Midwives in Sri Lanka) and recipients (patients), based on their cognitive and emotional responses to the components in the intervention [13]. The content, context and quality of care, each, has implications on the acceptability of the intervention [13].

The conceptual framework used in this study was the Theoretical Framework of Acceptability (TFA) which comprises of seven component constructs: affective attitude, burden, perceived effectiveness, ethicality, intervention coherence, opportunity costs, and self-efficacy, as shown in Box 1 [13].

## Box 1. The Theoretical Framework of Acceptability constructs and their definitions

**Table:** 

**Acceptability Concept**	**Definition**
**Affective attitude**	How an individual feel about the intervention
**Burden**	The perceived amount of effort that is required to participate in the intervention
**Ethicality**	The extent to which the intervention has a good fit with an individual’s value system
**Intervention coherence**	The extent to which the participant understands the intervention and how it works
**Opportunity costs**	The extent to which benefits, profits or values must be given up to engage in the intervention
**Perceived effectiveness**	The extent to which the intervention is perceived as likely to achieve its purpose
**Self-efficacy**	The participant’s confidence that they can perform the behaviour(s) required to participate in the intervention

### Study design

This was a multi-country, cluster randomized trial involving 30 communities (villages) in rural areas of Bangladesh, Pakistan, and Sri Lanka, stratified by country.

We have described the case settings previously in pre-implementation assessment of access to hypertension care services in the three countries [14]. A brief description is as follows:

#### Study setting

*Bangladesh*. Bangladesh is a low-middle- income country with a per-capita GDP of $1904, and a total population of 163 million, 63% is rural, and a literacy rate of 74% [15]. Prevalence of hypertension is 27%, with less than one-third controlled (BP <140/90 mm Hg) [11, 16]. The Bangladesh Health Assistants provide door to door maternal and childcare and immunization services. However, there is no mandate for NCD or hypertension care services by CHWs (Health Assistants). The sub districts, called Upizilla Health Complexes (UHCs) have recently established “NCD corners”; however, they are ill equipped and not adequately staffed. Although the drug formularies contain anti-hypertensive medications, they are not free of cost to the patients beyond 2- week supply.

*Pakistan*. Pakistan is a low-middle- income country with a per-capita GDP of $1590, and a total population of 216 million, 64% is rural, and a literacy rate of 59%. The prevalence of hypertension is 18% and 33% in those aged 18, and 45 or older, respectively, and less than 10% have controlled (BP <140/90 mm Hg) [17, 18]. The Lady Health Workers (LHW) provide door to door maternal and child care and immunization services. However, there is no mandate for NCD or hypertension care services by LHWs. The government’s basic health units are ill equipped with supplies and do not stock anti-hypertensive medications other than those for emergency management of pre-eclampsia and pregnancy-induced hypertension.

*Sri Lanka*. Sri Lanka is a middle- income country with a per-capita GDP of $4108 and a total population of 21.8 million, 81% is rural, and a literacy rate more than 90%. The prevalence of hypertension is 24%, with less than one-third with controlled (BP <140/90 mm Hg) [19]. The Public Health Midwives (PHMs) of Sri Lanka provide door to door maternal and childcare and immunization services. However, there is no mandate for NCD or hypertension care services by PHMs. The government clinics dispense antihypertensive medications, and the major antihypertensive medication classes are generally available, although the supplies run out sporadically. Only one-month supplies are dispensed at a single visit.

#### Sampling strategy

This qualitative study is embedded within a larger multi-country, cluster randomized trial involving 30 communities (villages) in rural areas of Bangladesh (districts of Tangail and Munshiganj, Pakistan (district Thatta), and Sri Lanka (district Puttlam). In each country, the unit of randomization was a cluster defined by 250–300 households defined by local administration according to CHW catchment area (each cluster of households served by one to two CHWs). The post- implementation qualitative study was undertaken in 5 intervention clusters in each country that were randomized to multicomponent intervention versus usual care, as explained in the COBRA-BPS protocol paper [20]. This paper is reported according to the Standards for Reporting Qualitative Research (SRQR) (**S1 Fig**).

The sampling strategy was purposive and based on sex, age-group, and those who had already been recruited in the trial in randomly selected clusters.

The Ethics Review Committees at icddr,b, Bangladesh (reference PR-15104), Aga Khan University, Pakistan, (reference 3833-CHS-ERC-15), University of Kelaniya, Sri Lanka (reference p/179/11/2015), London School of Hygiene & Tropical Medicine, UK (reference 10361), and Duke-NUS Medical School, Singapore (reference B-15-253) approved the study. The trial is registered at ClinicalTrials.gov. NCT02657746. The authors vouch for the completeness and accuracy of data, and the fidelity of the trial as reported.

#### Procedures

The interview guides were developed (by THJ, IJ, AdeS, AN, HLQ), pre-tested using probes and open-ended questions that were modelled on TFA to solicit the participants’ experienced acceptability of hypertension care in rural communities. A qualitative expert (HLQ) from Duke-NUS Singapore trained the site PIs at the icddr,b, Bangladesh, Aga Khan University, Pakistan, and University of Kelaniya, Sri Lanka, and all study coordinators in the common study protocol, qualitative methods, and principles of ethical research, and in using uniform probes modified to suit the local context. The guide covered opinions about experience, fit and acceptability of each component of the intervention, as relevant to the participants on: training of CHW and physicians, blood pressure monitoring and home health by CHWs, referrals to trained physicians using checklists and using treatment algorithm, hypertension triage counters and care coordinators at the government clinics, and compensation to CHW for additional services. This study allowed to understanding patients’ and healthcare providers’ attitudes on the acceptability and benefits of the intervention as well as their keenness on future participation which is important for long-term sustainability of the intervention and ultimately its effectiveness on reducing BP and cardiovascular morbidity and mortality.

The in-depth interviews of healthcare providers were conducted at the primary healthcare centers (their duty stations), and patients’ interviews were conducted in the respondents’ home after a pre-scheduled appointment. A written informed consent was obtained for the interviews and audio-recordings. Interviews were conducted by trained bilingual data collectors or researchers in local languages (Bangla, Urdu, Sindhi, and Sinhalese), and audio-recorded, transcribed, and then translated in English. The transcripts in Bangla and Sinhalese were translated into English and rechecked by the site PI and study coordinator. The transcriptions were done directly in English in Pakistan. All transcripts were reviewed line by line and double-checked for accuracy by the site PI (AdeS, AN, IJ) and study coordinators (CdeS, NC, SS) jointly for any disagreement. The final transcripts from each country were used for analysis. The audio-recordings and interview notes were consulted for validation purpose and consensus, when necessary.

#### Analysis

A code list was developed by THJ, ST AdeS, AN and IJ based on the objectives of the study.

The finalised transcripts were uploaded in NVivo version 11 software. The respective study interview guide themes were used for coding using inductive and thematic approach.

Coded verbatims were extracted through the analysis software by the research team members (CdeS, NC, MH) at each site, and checked by the site principal investigator (AdeS, AN, IJ). The code summaries were then interpreted thematically under the broader themes using framework analysis and grouped into the following TFA domains: 1) Affective attitudes: How healthcare providers and hypertensive individuals feel about COBRA-BPS intervention, 2) Burden: Challenges in community and healthcare system towards intervention implementation, 3) Ethicality: How COBRA-BPS has a good fit with healthcare system and community, 4) Intervention coherence: Understanding of intervention by hypertensive individuals and healthcare providers 5) Opportunity costs: Benefits, profits, or values that healthcare providers and hypertensive individuals must give up to engage in the intervention, 6) Perceived effectiveness: How the multicomponent intervention is perceived as likely to achieve program endpoints, 7) Self-efficacy: Hypertensive patients’ confidence to do behavioral changes and healthcare providers to perform interventions properly.

An additional theme of stakeholders’ views on scaling-up COBRA-BPS was also identified and analyzed. All represented quotes contained country name (BD- Bangladesh, PK-Pakistan, SL- Sri Lanka), participant type, and interview number. Participants’ names were replaced with pseudonyms, and quotes were de-identified to maintain data privacy. Participants’ age range, respondent type (patient, CHW, physician) and study location were retained. The summary analysis grouping was reviewed in depth by ST and chief PI (THJ), and any discrepancies were resolved after discussion with the relevant site PI and the respective site team (CdeS, MH, NC).

## Results

A total of 87 participants; 23 CHWs, 19 physicians and 45 hypertensive patients, participated in post intervention interview from the 15 intervention communities in rural Bangladesh, Pakistan and Sri Lanka. The interviews were conducted during 2016 to 2017 in Pakistan and to 2018 to 2019 in Bangladesh and Sri Lanka. The characteristics of the participants are shown in Table 1.

**Table 1:** Characteristic of participants.

Characteristics of Health Care Providers	Female	Male	Total
	N (%)	N (%)	N (%)
Sex	22	20	42
**Age range**			
30–39 Years	14 (63%)	8 (40%)	22 (52%)
40–49 Years	8 (36%)	10 (50%)	18 (42%)
50+ Years	-	2 (10%)	2 (5%)
**Providers type**			
Community Health Workers/PHM	20 (90%)	3 (15%)	23 (55%)
Physician	2 (10%)	17 (85%)	19 (45%)
**Location**			
Bangladesh	3 (13%)	7 (35%)	10 (24%)
Pakistan	5 (23%)	7 (35%)	12 (28%)
Sri Lanka	14 (64%)	6 (30%)	20 (48%)
**Characteristics of Patients**	**Female**	**Male**	**Total**
	**N (%)**	**N (%)**	**N (%)**
Sex	27	18	45
**Age range**			
40–49 Years	6 (22%)	2 (11%)	8 (18%)
50+ Years	21(78%)	16 (89%)	37 (82%)
**Location**			
Bangladesh	7 (26%)	9 (50%)	16 (36%)
Pakistan	7 (26%)	2 (11%)	9 (20%)
Sri Lanka	13 (48%)	7 (39%)	20 (44%)

The age range of the healthcare providers (CHWs and physicians) was 30 to 65 years, and of hypertensive patients was 40 to 70 years.

CHWs and hypertensive individuals reported that prior to COBRA-BPS multicomponent intervention they had never received hypertension management programs in their communities.

**Fig 1:**
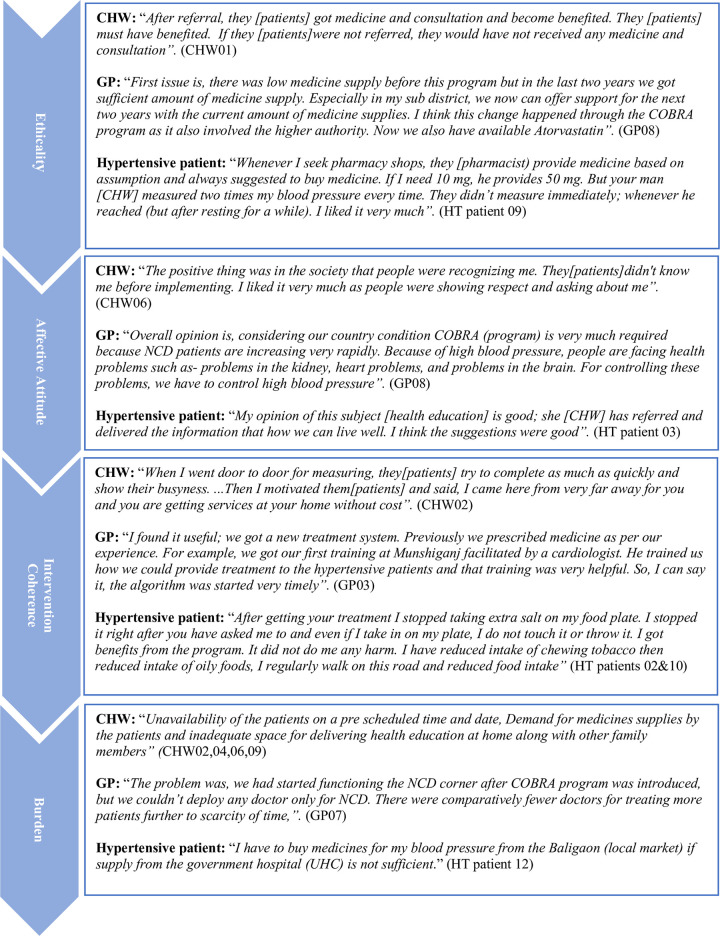
Illustrative quotes from Bangladesh participants.

**Fig 2:**
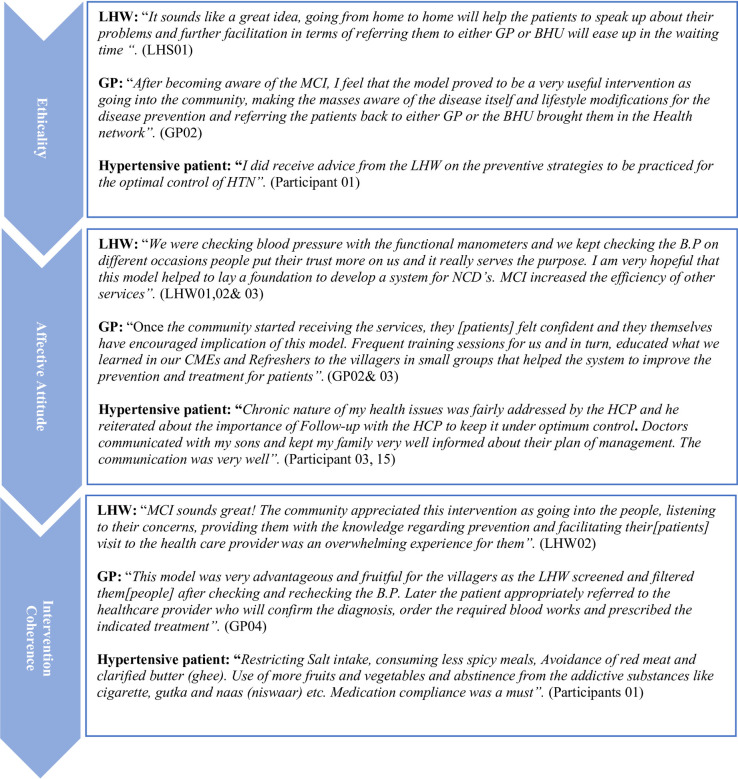
Illustrative quotes from Pakistan participants.

**Fig 3:**
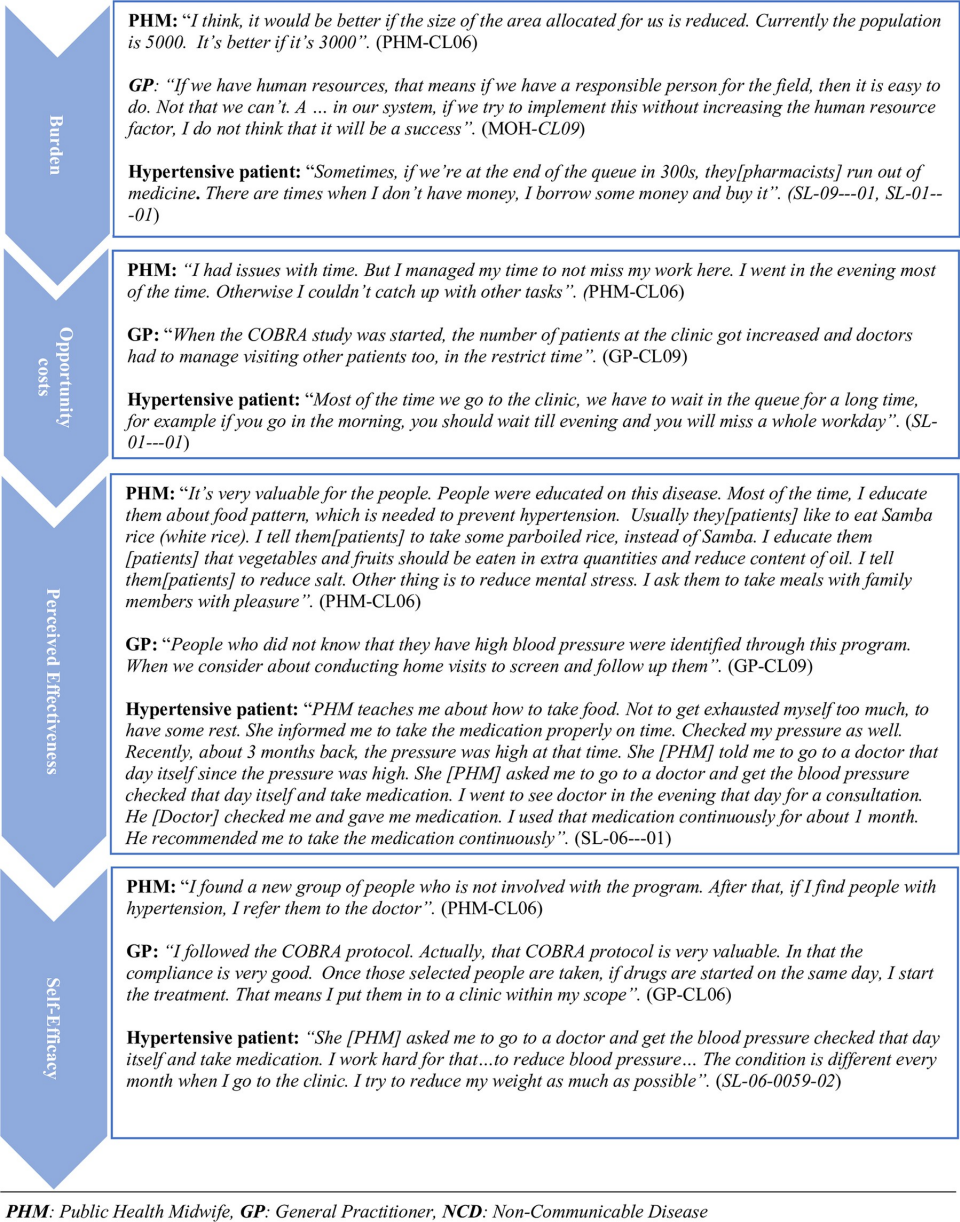
Illustrative quotes from Sri Lanka participants.

Figs 1–3 show the illustrative quotes under each TFA domain and study themes by the respondent type for each country.

### Ethicality

All CHWs, physicians and hypertensive patients in all 3 countries stated that the multicomponent intervention program was well within their healthcare system’s values, norms and beliefs. Majority of CHWs emphasized that intervention facilitated the identification of hypertensive patients in the community and their referral to the clinics for appropriate treatment. The following quote stated by one of LHWs in Pakistan:

“*It was a great program*, *going from home to home helped the patients to speak up about their problems and facilitated referring them to either GP or BHU (basic health unit) and eased up in the waiting time”*. (PK-LHS01)

All physicians believed that the program was well aligned with existing healthcare services in all 3 countries, and created the demand for high quality services. In their view, this seemed to be a consequence to more informed and activated patients who were exposed to standardized and efficient system of hypertension care delivery through their participation in the intervention. Below is the statement of a physicians in Sri Lanka:

“*At the NCD clinic or even at the OPD*, *initially a patient met a health work assistant*. *The health work assistant checked his BP then register him*. *If the pressure was very high*, *she referred the patient immediately*. *Sometimes she [PHM] came with a patient that his pressure is high*. *If blood pressure was not very high but it is above 140/90*, *although it was not urgent because it was still high*, *we asked them[patients] to be in the queue and visit them on the same day*. *If it was not high*, *we asked them to come back and check their pressure again*. *After checking their BP for three times*, *we decided whether they[patients] require to register in the clinic or not”*. (GP-SL-CL09)

All hypertensive patients described their interest in keeping healthy in all 3 countries; thus, the intervention complemented their beliefs as they are receiving optimal control of hypertension, and it is accepted by the community and themselves. One of the patients in Bangladesh expressed her ideas as below:

“*Whenever I sought pharmacy shops*, *they [pharmacist] provided medicine based on assumption and always suggested to buy medicine*. *If I needed 10 mg*, *he gave me 50 mg*. *But your man [CHW] measured two times my blood pressure every time*. *He didn’t measure immediately whenever he reached (but after resting for a while)*. *I liked it very much”*. (BD-HT Patient 09)

#### Affective attitude

Most of CHWs supported the intervention and expressed willingness to continue to participate in its delivery. CHWs believed that the care they delivered during home visits- BP measurements, and identifying hypertensive patients, home health education, and referrals—contributed to better NCD management in the community. Additionally, CHWs noted that the intervention increased people’s trust and respect towards them, and the hypertension care services they delivered filled a large gap in healthcare system in the rural areas. As one of the PHMs in Sri Lanka described it well below:

*“The program conducted by COBRA*, *to guide us towards hypertension management was a very special program*. *Furthermore*, *we generally direct the community towards NCD management*. *The program was truly good*. *We went to rural places looking for patients to and find patients and treat them*. *Other programs are not like that*. *Patients have to go looking for such services*. *In COBRA program*, *we were not like that*. *We went to the community and identified patients who needed treatments*. *When the program was started*, *we went to their house and checked their (blood) pressure and asked if they[patients]were taking medicines… and about their food habits*, *and gave them[patients] advice… That’s how we dealt with hypertension”*. (SL-PHM-CL06)

From another perspective, the patients valued receiving education on hypertension and measures to prevent and control. Thus, they are willing to participate in the programme. Following quote is from one of the patients in Bangladesh:

*“My opinion of this subject [health education] is good; she [CHW] referred and delivered the information that how we can live well*. *I think the suggestions were good”*. (BD- HT Patient 03)

Moreover, most CHWs and physicians in three countries reported that implementing multicomponent intervention with an outreach of hypertension care services to patients’ doorsteps increased the rural healthcare sector’s efficiency. They opined that lifestyle advice enabled people to realize their unhealthy eating habits and guided them toward improving BP control behaviour. Moreover, the compensation given to CHWs for additional services related to intervention delivery greatly facilitated fidelity. Most CHWs believed that a great advantage of the COBRA model program was that it obviated people’s need to commute long distances to seek medical care for BP monitoring, and therefore reduced travel costs for the patients. Furthermore, most physicians thought that hypertension triage counter and care coordinators in clinics were needed to reduce waiting time. The physicians were also appreciative of the training they received in updated guidelines for managing hypertension and noted that training programs of this kind did not exist before. The next quotes from physicians in Pakistan and Sri Lanka illustrate examples of such instances:

*“HTN Triage counter and HTN care coordinators in Government Clinics were very much needed in order to curtail the waiting time faced by the patients*. *It could decrease the workload of the doctors and reduce the waiting hours as well”*. (PK-GP06)

*“(*Previously*) we did not follow any guideline in prevention and management (of hypertension)*. *Once the COBRA-BPS study was started*, *hypertensive patients were managed according to the guidelines provided by the COBRA-BPS”*. (SL-GP-CL09)

#### Intervention coherence

Most CHWs and physicians expressed a good understanding of the multicomponent intervention, including home health education and easy-to-use hypertension treatment based on the study algorithm and described it well when asked. Some CHWs and physicians explained every step of the care delivery process. Here is the statement of a physician from Pakistan: “*This model sounds to be very advantageous and fruitful for the villagers as the LHW screened and filtered them[people] after checking and rechecking the B*.*P*. *Later the patient appropriately referred to the healthcare provider who would have confirmed the diagnosis*, *ordered the required blood samples and prescribed the indicated treatment based on study algorithm”*. (PK-GP04)

Many hypertensive patients expressed understanding of the components of program as they reported changes in behaviors in response to HHE including restricting salt intake, avoidance of red meat and clarified butter, consuming more fruits and vegetables, “mallum” and avoidance of addictive substances like cigarettes, alcohol, “gutka” and “niswaar”, coconut oil and adherence to anti-hypertensive medications. The following is a quote from participants in Bangladesh who reported how they changed their harmful habits to good healthy ones:

“*After getting your treatment I stopped taking extra salt on my food plate*. *I stopped it right after you asked me to and even if I took in my plate*, *I did not touch it or threw it*. *I got benefits from the program*. *It did not do me any harm*. *I reduced intake of chewing tobacco then reduced intake of oily foods*, *I regularly walk on this road and reduced food intake”* (BD- HT Patients 10&02)

#### Burden

Some CHWs in all 3 countries mentioned challenges related to intervention delivery such as transport, big areas of coverage, increased workload, and lack of provision of medications. Regarding the door-to-door home visit, few CHWs noted that they faced challenging situations with uncooperative households who did not welcome them in their homes on a few occasions. The following quote from a PHM in Sri Lanka is an example:

*“certain people were used to hide upon the arrival of the CHW*. *Sometimes*, *when I went to check the husband*, *the wife came and lied about the availability of the husband”* (SL-PHM01-CL07)

From physicians’ perspective, some of them reported insufficient personnel, whilst they had to cover a large number of patients in clinic which was burdensome. Following is the statement of one of physicians in Bangladesh”

“*The problem is*, *we started functioning the NCD corner after COBRA program was introduced*, *but we couldn’t deploy any doctor only for NCD*. *Here we treat all types of patients including NCD*. *So*, *the time required for treating NCD patients is taken another patients time*. *There is comparatively fewer doctors for treating more patients”*. (BD-GP07)

The common barriers highlighted by many hypertensive patients in three countries were shortage of medicine, and low affordability of buying medicine. Here is a quote how patients in Sri Lanka reported the barrier:

*Sometimes*, *if we were at the end of the queue of about n 300s*, *they[pharmacists] ran out of medicine*. *There were times when I had to borrow money to buy those medicines as I did not have (money)”*. (SL-09—01, SL-01—01)

Few patients mentioned they could not afford or not have access the healthy foods like fruits and vegetables as stated below by a patient from Bangladesh:

“*I couldn’t follow her [CHW] dietary*. *For example- today*, *I am working in managing paddy and it is not possible to go to the market*. *We take lunch by any means which are available at home*. *It may be potato or banana mash or fry*.*”* (BD-HT patient10)

#### Opportunity costs

Among CHWs, some mentioned reduced time for other routine activities, as they had to accommodate the intervention delivery along with their routine tasks. As a PHM in Sri Lanka mentioned:

*“I had issues with time*. *But I could manage my time without missing to not miss my work here*. *I went (to the field to do home visits) in the evening most of the time*. *Otherwise I couldn’t catch up with other tasks”*. (SL*-*PHM-CL06)

Majority of physicians mentioned that they spent more time for treatment of hypertensive patients based on intervention treatment algorithm. Some physicians voiced that waiting time for other patients in their clinics increased.

Hypertensive patients reported that their attendance in clinic for their treatment follow up affected the time they spend working as stated in the following quote from a patient in Sri Lanka:

“*Most of the time we went to the clinic*, *we had to wait in the queue for a long time*, *for example one day I went in the morning*, *and I had to wait till evening*. *So*, *I missed a whole workday”*. (*SL-01—01*)

#### Perceived effectiveness

The majority of CHWs and physicians stated that the intervention positively influenced patients’ knowledge, food habits, and physical activity. They were especially appreciative of CHW training as the prior practices were considered outdated. In their view, home health education helped people learn and practice the essential facts for the prevention and control of hypertension. As described by healthcare providers in Bangladesh and Sri Lanka:

“*It [health education] was benefited to the patients because they didn’t know about health education prior*. *Before the program they [patients] ate extra salt*, *now they don’t*. *They [patients] ate betel leaf*, *chewing tobacco now they don’t*, *they didn’t walk*, *now they do*. *Now their [patients] blood pressure is under control*”. (BD-CHW11)

*People who did not know that they have high blood pressure were identified through this program*, *while we considered about conducting home visits to screen and follow up them”*. (SL*-*GP-CL09)

When asked about how the intervention was effective and improved management of hypertension in the community, majority of physicians believed that this model of multicomponent intervention with home health education and home BP monitoring by CHW coupled with training of physicians led to more activated patients and well trained physicians, and ultimately resulted in improved BP control. The following quote summarizes the sentiments of those physicians stated by a physician in Pakistan:

*“Once the community started receiving the services*, *they [patients] felt confident and they themselves encouraged implication of this model*. *By frequent training sessions*, *CMEs and refresher training*, *in turn we gave what we learned to the villagers in small groups*, *so helped improvement of prevention and treatment of hypertension”*. (PK-GP02)

Some physicians mentioned that particular measures were indicative of increased effectiveness of the intervention in a rural setting, and patients’ dietary habits and adherence to antihypertensive medications were improving. Moreover, many patients reportedly stopped using alternative medicine or bought over-the-counter from pharmacies and switched to physician-prescribed medications. They also tried to reduce dietary salt and saturated fat intake. Physicians found that patients to be better informed about the risks and complications of hypertension. As reported by a physician in Bangladesh:

“*They [patients] were benefited*. *Because the patients*, *who didn’t visit the doctors before*, *they visited and collected medicine from the UHC following the program regularly*. *They[patients] observed some positive changes and understand that the medicine should be taken for a lifetime on a regular basi*s”. (BD- GP-03)

Several hypertensive patients reported that home health education was beneficial for them and their families. They underscored that several people in the community were also willing to participate in the intervention. They stated that their lifestyle changed positively by participating in this program, and they adhere to their medicine rigorously. The following is a quote from a patient in Sri Lanka: “*PHM taught me about how to take food*. *Not to get exhausted myself too much*, *to have some rest*. *She informed me to take medications properly on time*. *Checked my pressure as well*. *Recently*, *about 3 months back*, *the pressure was high at that time*. *She [PHM] told me to go to a doctor that day itself since the pressure was high*. *She [PHM] asked me to go to a doctor and get the blood pressure checked that day itself and take medication*. *I went to see a doctor in the evening that day*. *He [Doctor] checked me and gave me medication*. *I used that medication continuously for about 1 month*. *He recommended me to take the medication continuously”*. (SL-06—01)

#### Self-efficacy

Many CHWs reported that they became confident in their ability to deliver the intervention and achieve the desired results. In their view, the outstanding training they received during the intervention empowered them to detect hypertension. They reported their high level of ability to deliver HHE advice to the people in the community. An LHW in Pakistan mentioned:

“*As we were adequately trained regarding the dietary modifications and know what we were supposed to educate the patient and it was very beneficial for the patient as our training was really outdated”*. (PK*-*LHW03)

Several CHWs mentioned that respect shown by the patients to trained healthcare providers as one of the foremost reasons for their enthusiasm to continue to participate in the program. Majority of physicians revealed very positive attitude regarding BP measurement and referral activities done by CHWs. They also showed their satisfaction about the process of screening, visiting hypertensive patients in clinic and regular follow-ups. As described by a physician in Sri Lanka:

*“I followed the COBRA protocol*. *Actually*, *that COBRA protocol was very valuable*. *In that the compliance of patients was very good*. *Once those selected people were registered*, *I started the medications for them on the same day*. *That means I put them in clinic within my scope”*. (SL-GP-CL06)

Most hypertensive patients in the three countries expressed happiness at becoming more knowledgeable about hypertension and recognized the importance of adherence to blood pressure-lowering medications. Indeed, they mentioned that they changed their lifestyle for good as they followed the advice from CHWs and adopted the suggested behaviors accordingly. The next quote from a patient in Sri Lanka is an example of the many reports in the three countries:

*“PHM taught me about how to take food*. *She asked me to go to a doctor and get the blood pressure checked that day itself and take medication*. *I work hard for that…to reduce blood pressure… I get my blood pressure checked every month when I went to the clinic for follow up*. *I also try to reduce my weight as much as possible”*. (SL-06—02*)*

#### Perceived usefulness

The vast majority of healthcare providers (CHWs and Physicians) and hypertensive patients found the COBRA intervention useful and voiced a need for continuation of the same in the in their community. CHWs were enthusiastic to continue the program, and screen even those individuals for high blood pressure who were not in the study. They faced an increase in demands for BP monitoring and HHE from family members and other community dwellers. One of CHWs from Bangladesh mentioned:

*“Our country people want to check their blood pressure*. *There were a lot of people who demanded to check BP*. *They are other family members of patients and their neighbors*, *it[program] needs to cover more people”* (BD-CHW-01,09)

Most physicians also alluded that their country needs such a program to screen and manage hypertension. They knew that plenty of hypertensive patients were unaware of their hypertension status and have never had their blood measured. Therefore, the COBRA program would serve them well. A physician in Bangladesh highlighted the need for such a program in the community:

*“It (BP monitoring) is a good system*. *It can reduce the patient load at the health complex because it is not possible to check every patient blood pressure*. *Community health workers monitored blood pressure and provided health education to the patients which were so beneficial*. *I think the system is good and such screening system is required in the community”* (BD-GP07)

Many physicians expressed that the hypertension treatment algorithm will be useful for all hypertensive patients. They seemed to understand the implementation of COBRA multicomponent intervention as routine care will lead to the standardized treatment of patients using an evidence-based systematic method and, therefore, is likely to be more effective and efficient at achieving BP control. The following quote mentioned by a physician from Pakistan:

*“We are only catering the patient population presented to us*. *Plenty in the community are untouched*. *It will be a more than welcome if the program deploys to all population*, *surely it will improve the dynamics of this health condition and overall management of HTN on the basis of guideline*.” (PK-GP05,07)

Most of the hypertensive patients were grateful of being included in the program. They appreciated the knowledge and awareness they gained from the intervention on healthy lifestyle to better manage their BP. The next quote from a hypertensive patient in Sri Lanka illustrates example of such expressions:

*“The doctor checks the blood pressure and prescribes medicine*. *They ask us to come back again to check blood pressure*. *Only now we know regarding the things you taught us*. *Basically*, *regarding what we should do to manage hypertension*. *Therefore*, *this is a very valuable program”*. (SL-06—-02)

## Discussion

We report findings from 87 post-implementation interviews of patients, CHWs, and physicians assessing COBRA-BPS multicomponent intervention strategies’ acceptability in rural Bangladesh, Pakistan, and Sri Lanka at the health systems level using the theoretical framework of acceptability [13]. Based on all stakeholders’ feedback in three countries, the critical components of COBRA-BPS multicomponent intervention- trained CHW linked with an enhanced public health system with trained physicians- had a good fit and aligned well with the values of the respective health systems. In all three countries, most patients and healthcare providers had a good understanding of all components of COBRA-BPS multicomponent intervention. The CHWs felt more empowered and were enthusiastic about continuing despite some challenges in reaching very remote households. The program’s perceived effectiveness on patients’ behaviour and their admiration and respect for trained CHW and trained physicians were considered primary facilitators. Most patients felt more knowledgeable about lifestyle changes to control BP because of better education by trained CHW and trained physicians, valued home BP monitoring, and enhanced services. Most physicians appreciated that the patients trusted them more, and the intervention was useful, and patients’ were more adherent to the prescribed antihypertensive medications. The lack of free medications was perceived as the most problematic aspect of the program by the patients and CHWs, especially in Bangladesh and Pakistan. All stakeholders who participated considered multicomponent intervention to be a valuable program that filled an unmet need for hypertension care in the rural communities, and favoured scaling-up at a national level. We strongly encourage further community engagement to explore scaling-up COBRA-BPS in rural communities in Bangladesh, Pakistan, and Sri Lanka and other LMICs with similar healthcare infrastructure.

Previous evaluations of CHW-led NCD interventions’ acceptability and their barriers and facilitators were mainly limited to pre-implementation studies [7, 21]. Previously, we identified several barriers to accessing health services for hypertension management, as reported by participants in 3 countries, including doctors’ busyness, long transportation, long waiting times, and medication shortages. Our current post-intervention assessment of participants revealed that COBRA-BPS multicomponent intervention appeared to address these challenges. Patients in all three countries were appreciated better communication with the physicians, and the BP monitoring services and home health education provided by CHWs in their homes. Our findings underscore that the physicians and CHWs felt rewarded in a manner that was meaningful to them. For example, the physicians reported feeling more trusted by the patients, which was corroborated by the patients in all three countries. Evidence suggests that interventions that reduce clinician-patient miscommunication are more likely to be successful in influencing patient behaviours on lifestyle, adherence to medications, and ultimately cardio-metabolic disease control [22, 23]. Furthermore, patients in Bangladesh and Pakistan reported increased utilization of the public health sector. They changed medications purchasing behaviour to those prescribed by the physicians instead of obtaining over-the-counter at the private pharmacy, as practiced before the intervention [23]. Of note, antihypertensive medication treatment intensification and adherence to antihypertensive medications were more significant in the intervention compared to usual care participants in the trial, indicating a favourable association of the mutual trust and confidence between patients and providers trained in the intervention [14].

Consistent with the feasibility study report, the CHWs in all three countries felt more respected and empowered in the communities and felt motivated to deliver high-quality care [9]. Increased workload due to the COBRA-BPS multicomponent intervention was highlighted as a challenge by the CHWs. This is understandable as BP monitoring and HHE are not part of the routine maternal and child health services provided by CHWs [24]. However, the compensation for additional hypertension care services in COBRA-BPS multicomponent intervention were considered a major facilitator [24]. Moreover, since the payments in COBRA-BPS multicomponent intervention were channelled through the district health system, the mechanism provides the opportunity to hire additional staff to redistribute work, especially when scaled-up. Of note, the compensation for additional services were accounted for in the budget impact analysis for scale-up of COBRA-BPS multicomponent intervention, which yielded a cost of less than $2 per-capita annually, and therefore qualifies as a WHO best-buy intervention for achieving a 30% reduction in premature mortality from NCDs by 2030 [25–27].

However, we also found some persistent health systems challenges, which, if addressed, could further enhance the acceptability of our COBRA-BPS multicomponent intervention. Of note, the lack of provision of antihypertensive medications free of cost in the primary care clinics was a frequent complaint expressed by the patients [3, 14, 28]. High out-of- pocket cost of antihypertensive medications is a known barrier to BP control in many South Asian countries [28]. The PURE study showed that 31% of households in low-income countries could not afford two blood pressure-lowering medicines [28]. The WHO Essential Drug list includes the major classes of antihypertensive medications. Although antihypertensive medications are free in Sri Lanka, our results show the problem of under stocking and long queues at the clinic being a deterrent to the accessibility of medications to the patients. Studies in China report similar challenges with accessibility to antihypertensive medications [29]. Our findings underscore the need to ensure generic low-cost antihypertensive medications at the primary care facilities and their accessibility to patients in rural South Asia and many other countries globally. Our study has several strengths. First, ours is one of the few post-intervention acceptability studies on hypertension management strategies in South Asia. It has tremendous implications for scaling-up practical, effective, cost-effective, and affordable intervention. Second, the representation of different stakeholders, including cadres of health workers and patients in the health system, enhances our findings’ validity. Third, the inclusion of three rural LMICs enhances our findings’ generalizability to other LMICs, especially in the rural sector. Fourth, the theory of acceptability is a robust framework for analysis.

Our study has some limitations. First, the timing of the interviews was after the completion of the trial in Bangladesh and Sri Lanka (2019), and during the course of the trial in Pakistan (2016–2017), albeit after the intervention was implemented in all three countries in 2016. Second, more participants were recruited from Sri Lanka, compared to the Bangladesh or Pakistan, each. However, the country and patient demographics, and health systems of Pakistan and Bangladesh are quite similar and cumulatively they contributed the same number of participants as Sri Lanka, albeit not stratified in the subgroups as originally intended in the protocol. Community engagement efforts prior to scale-up must ensure seeking feedback from all representative subgroups especially in the more populous countries of Bangladesh and Pakistan. Moreover, the acceptability of district and national health managers and policymakers will need to be assessed.

In conclusion, our post-intervention assessment on 87 physicians, community health workers, and patients strongly suggest that the multicomponent COBRA-BPS multicomponent intervention is acceptable with a good fit and highly valued by the rural stakeholders Bangladesh, Pakistan, and Sri Lanka. Physicians and community health workers reportedly felt more rewarded despite an increase in workload, which they managed well. Patients’ improved adherence to physicians’ and CHWs’ advice and adopted healthy behaviour. Our findings of excellent acceptability coupled with effectiveness, co-effectiveness, and affordability, make a compelling case for further community engagement for scaling-up COBRA-BPS in the rural communities in Bangladesh, Pakistan and Sri Lanka and other LIMCs with similar healthcare infrastructure.

## Supporting information

S1 Fig Standards for Reporting Qualitative Research (SRQR). (PDF)

S1 File COBRA-BPS qualitative study protocol. (DOCX)

S2 File COBRA-BPS study team members list. (PDF)

S3 File Code list healthcare providers. (PDF)

S4 File Code list patients. (PDF)

S5 File Interview guide healthcare providers. (PDF)

S6 File Interview guide patients. (PDF)

## References

[1] Shah AD, Kandula NR, Lin F, Allison MA, Carr J, Herrington D, et al. Less favorable body composition and adipokines in South Asians compared with other US ethnic groups: results from the MASALA and MESA studies. Int J Obes (Lond). 2016;40(4):639–45. doi: 10.1038/ijo.2015.219 26499444 PMC4821815

[2] Mills KT, Bundy JD, Kelly TN, Reed JE, Kearney PM, Reynolds K, et al. Global Disparities of Hypertension Prevalence and Control: A Systematic Analysis of Population-Based Studies From 90 Countries. Circulation. 2016;134(6):441–50. doi: 10.1161/CIRCULATIONAHA.115.018912 27502908 PMC4979614

[3] Jafar TH, Gandhi M, Jehan I, Naheed A, de Silva HA, Shahab H, et al. Determinants of Uncontrolled Hypertension in Rural Communities in South Asia-Bangladesh, Pakistan, and Sri Lanka. Am J Hypertens. 2018;31(11):1205–14. doi: 10.1093/ajh/hpy071 29701801 PMC6188532

[4] Misra A, Tandon N, Ebrahim S, Sattar N, Alam D, Shrivastava U, et al. Diabetes, cardiovascular disease, and chronic kidney disease in South Asia: current status and future directions. BMJ. 2017;357:j1420. doi: 10.1136/bmj.j1420 28400361

[5] Yusuf S, Rangarajan S, Teo K, Islam S, Li W, Liu L, et al. Cardiovascular risk and events in 17 low-, middle-, and high-income countries. N Engl J Med. 2014;371(9):818–27. doi: 10.1056/NEJMoa1311890 25162888

[6] Jafar TH, Gandhi M, de Silva HA, Jehan I, Naheed A, Finkelstein EA, et al. A Community-Based Intervention for Managing Hypertension in Rural South Asia. N Engl J Med. 2020;382(8):717–26. doi: 10.1056/NEJMoa1911965 32074419

[7] Legido-Quigley H, Camacho Lopez PA, Balabanova D, Perel P, Lopez-Jaramillo P, Nieuwlaat R, et al. Patients’ knowledge, attitudes, behaviour and health care experiences on the prevention, detection, management and control of hypertension in Colombia: a qualitative study. PLoS One. 2015;10(4):e0122112. doi: 10.1371/journal.pone.0122112 25909595 PMC4409332

[8] Naheed A, Haldane V, Jafar TH, Chakma N, Legido-Quigley H. Patient pathways and perceptions of hypertension treatment, management, and control in rural Bangladesh: a qualitative study. Patient Prefer Adherence. 2018;12:1437–49. doi: 10.2147/PPA.S163385 30147302 PMC6097513

[9] Jafar TH, Silva A, Naheed A, Jehan I, Liang F, Assam PN, et al. Control of blood pressure and risk attenuation: a public health intervention in rural Bangladesh, Pakistan, and Sri Lanka: feasibility trial results. J Hypertens. 2016;34(9):1872–81. doi: 10.1097/HJH.0000000000001014 27488552

[10] Perera M, de Silva CK, Tavajoh S, Kasturiratne A, Luke NV, Ediriweera DS, et al. Patient perspectives on hypertension management in health system of Sri Lanka: a qualitative study. BMJ Open. 2019;9(10):e031773. doi: 10.1136/bmjopen-2019-031773 31594895 PMC6797394

[11] Chowdhury MA, Uddin MJ, Haque MR, Ibrahimou B. Hypertension among adults in Bangladesh: evidence from a national cross-sectional survey. BMC Cardiovasc Disord. 2016;16:22. doi: 10.1186/s12872-016-0197-3 26809175 PMC4727356

[12] Davidoff F, Dixon-Woods M, Leviton L, Michie S. Demystifying theory and its use in improvement. BMJ Qual Saf. 2015;24(3):228–38. doi: 10.1136/bmjqs-2014-003627 25616279 PMC4345989

[13] Sekhon M, Cartwright M, Francis JJ. Acceptability of healthcare interventions: an overview of reviews and development of a theoretical framework. BMC Health Serv Res. 2017;17(1):88. doi: 10.1186/s12913-017-2031-8 28126032 PMC5267473

[14] Legido-Quigley H, Naheed A, de Silva HA, Jehan I, Haldane V, Cobb B, et al. Patients’ experiences on accessing health care services for management of hypertension in rural Bangladesh, Pakistan and Sri Lanka: A qualitative study. PLoS One. 2019;14(1):e0211100. doi: 10.1371/journal.pone.0211100 30682093 PMC6347162

[15] world bank group. Bangladesh country profile.https://data.worldbank.org/country/bangladesh. 2016.

[16] Rahman M, H SE, Islam MJ, Mostofa MG, Saadat KA. Association of socioeconomic status with diagnosis, treatment and control of hypertension in diabetic hypertensive individuals in Bangladesh: a population-based cross-sectional study. JRSM Open. 2015;6(10):2054270415608118. doi: 10.1177/2054270415608118 26688743 PMC4601127

[17] Jafar TH, Levey AS, Jafary FH, White F, Gul A, Rahbar MH, et al. Ethnic subgroup differences in hypertension in Pakistan. J Hypertens. 2003;21(5):905–12. doi: 10.1097/00004872-200305000-00014 12714864

[18] Jafar TH, Haaland BA, Rahman A, Razzak JA, Bilger M, Naghavi M, et al. Non-communicable diseases and injuries in Pakistan: strategic priorities. Lancet. 2013;381(9885):2281–90. doi: 10.1016/S0140-6736(13)60646-7 23684257

[19] Katulanda P, Ranasinghe P, Jayawardena R, Constantine GR, Rezvi Sheriff MH, Matthews DR. The prevalence, predictors and associations of hypertension in Sri Lanka: a cross-sectional population based national survey. Clin Exp Hypertens. 2014;36(7):484–91. doi: 10.3109/10641963.2013.863321 24433043

[20] Jafar TH, Jehan I, de Silva HA, Naheed A, Gandhi M, Assam P, et al. Multicomponent intervention versus usual care for management of hypertension in rural Bangladesh, Pakistan and Sri Lanka: study protocol for a cluster randomized controlled trial. Trials. 2017;18(1):272. doi: 10.1186/s13063-017-2018-0 28606184 PMC5469065

[21] Khatib R, Schwalm JD, Yusuf S, Haynes RB, McKee M, Khan M, et al. Patient and healthcare provider barriers to hypertension awareness, treatment and follow up: a systematic review and meta-analysis of qualitative and quantitative studies. PLoS One. 2014;9(1):e84238. doi: 10.1371/journal.pone.0084238 24454721 PMC3893097

[22] Lee YY, Lin JL. The effects of trust in physician on self-efficacy, adherence and diabetes outcomes. Soc Sci Med. 2009;68(6):1060–8. doi: 10.1016/j.socscimed.2008.12.033 19162386

[23] Jneid S, Jabbour H, Hajj A, Sarkis A, Licha H, Hallit S, et al. Quality of Life and Its Association With Treatment Satisfaction, Adherence to Medication, and Trust in Physician Among Patients With Hypertension: A Cross-Sectional Designed Study. J Cardiovasc Pharmacol Ther. 2018;23(6):532–42. doi: 10.1177/1074248418784292 29916266

[24] Jafar TH. A Community-Based Intervention for Hypertension in Rural South Asia. Reply. N Engl J Med. 2020;382(25):e99. doi: 10.1056/NEJMc2006112 32558482

[25] collaborators NC. NCD Countdown 2030: worldwide trends in non-communicable disease mortality and progress towards Sustainable Development Goal target 3.4. Lancet. 2018;392(10152):1072–88. doi: 10.1016/S0140-6736(18)31992-5 30264707

[26] Jafar TH, Islam M, Bux R, Poulter N, Hatcher J, Chaturvedi N, et al. Cost-effectiveness of community-based strategies for blood pressure control in a low-income developing country: findings from a cluster-randomized, factorial-controlled trial. Circulation. 2011;124(15):1615–25. doi: 10.1161/CIRCULATIONAHA.111.039990 21931077 PMC3192033

[27] Bertram MY, Sweeny K, Lauer JA, Chisholm D, Sheehan P, Rasmussen B, et al. Investing in non-communicable diseases: an estimation of the return on investment for prevention and treatment services. Lancet. 2018;391(10134):2071–8. doi: 10.1016/S0140-6736(18)30665-2 29627159

[28] Attaei MW, Khatib R, McKee M, Lear S, Dagenais G, Igumbor EU, et al. Availability and affordability of blood pressure-lowering medicines and the effect on blood pressure control in high-income, middle-income, and low-income countries: an analysis of the PURE study data. Lancet Public Health. 2017;2(9):e411–e9. doi: 10.1016/S2468-2667(17)30141-X 29253412

[29] Su M, Zhang Q, Bai X, Wu C, Li Y, Mossialos E, et al. Availability, cost, and prescription patterns of antihypertensive medications in primary health care in China: a nationwide cross-sectional survey. Lancet. 2017;390(10112):2559–68. doi: 10.1016/S0140-6736(17)32476-5 29102087

