# Supplementary material for: Post-intervention acceptability of multicomponent intervention for management of hypertension in rural Bangladesh, Pakistan, and Sri Lanka- a qualitative study
Source: PLoS One. 2023 Jan 19;18(1):e0280455. doi: 10.1371/journal.pone.0280455 (PMC9851540; doi:10.1371/journal.pone.0280455)
Supplement: S1 File — (DOCX) [file pone.0280455.s002.docx]

**Qualitative Sub-Study protocol**

**Objectives**

The specific objectives of the complementary qualitative study are:

1. To conduct a rapid literature review (a lot of the data already collected) and use it to inform an analysis of how hypertension is managed in each country’s health care system; the burden of disease for hypertension; how services for hypertension are organized and funded; and what hypertension prevention and treatment policies exist at country level.
2. To identify hypertensive individuals’ experiences of symptoms, awareness of prevention, knowledge and control of hypertension.
3. To explore the perceptions of hypertensive individuals, healthcare professionals, providers and policy makers on the perceived usefulness of the intervention, its impact on existing practice, challenges of incorporating the intervention and whether the intervention and compensation have affected other services.
4. To compare the perspectives and attitudes of hypertensive individuals, health care providers and policy-makers on the prevention, treatment and control of hypertension pre and post intervention.
5. To identify the health systems-level barriers and facilitators to the effective prevention, treatment and control of hypertension, from the perspective of the hypertensive individual, health care provider and policy-maker.

**Methods**

In each of the 15 intervention clusters (5 per country), interviewees will be undertaken at the level of the clusters included in the intervention study. Each country will recruit 20 hypertensive individuals based on their ethnic group, age, gender, hypertensive status (controlled, uncontrolled) and socio-economic status (SES). They will also interview 15 Health care Professionals type (provincial and district-level coordinators of the government community health workers’ programme; NCD coordinator or focal person appointed at the central and provincial levels and relevant appointees in finance/economic departments; physician in-charge of the basic health unit in each cluster; community health workers and their supervisors; midlevel providers and their supervisor; private practitioners; and key community leaders) in each country.

This study will involve conducting semi-structured interviews with hypertensive individuals, health care professionals, providers, and policy-makers. This project is framed as a micro study, using in-depth interviews as data generating instruments, while drawing on grounded theory techniques to develop understanding of the phenomena. The study will adopt thematic analysis of semi-structured interviews.

The respondents will be given an information sheet and asked to sign a consent form. Confidentiality was ensured by giving each participant the option of not being quoted, even anonymously, in the study and subsequent publications; and quoting participants without reference to their age, sex, professional status and role. To further protect the confidentiality of study participants, each participant will be assigned a unique Participant Identification Number (PID). Interview tapes and transcripts will be available to the study researchers only and be stored in a secured file. In the research paper, individual sub-groups were referred to only in general terms.

The post-intervention interviews will focus on exploring the differences in perceptions and attitudes on the prevention, treatment and control of hypertension; the perceived usefulness of implementing the care strategy; its impact on practice and other services; the overall satisfaction with referral set-up; and suggestions for improvement.

**Outcomes**

Feasibility of the full-scale trial depends on stakeholder attitudes toward integration of the proposed care strategies into the existing healthcare system. This component is critical in sustaining the proposed care strategies on a long-term basis. We anticipate that stakeholder perceptions of the usefulness of the care strategy and its impact on access to healthcare (for individuals) and work practice (for healthcare providers) are major factors influencing these attitudes. Thus, stakeholder experiences during the feasibility will be used to understand their attitudes toward the full-scale trial. Barriers and facilitators identified by stakeholders will be investigated by the study team, and stakeholders will be engaged to address potentially modifiable factors prior to the full-scale trial.

**Data collection & Analysis**

The interviews will be audio recorded and translated directly from the respective languages to English on transcripts by an expert bilingual, native speaker. The country research teams will then analyze the data thematically in two steps. The first step consists of a deductive analysis, coding units of data according to key inputs and other elements of the theoretical framework, which will inform the study design (e.g. human resources, funding, etc.). This is followed by an inductive analysis, seeking to elicit new themes or unexpected findings through coding and categorizing, following some of the techniques of grounded theory which include looking for deviant cases and using the constant comparison method by comparing codes and categories. Relevant quotes representative of the analysis will be incorporated into the publications. Data will be managed using QSR International’s NVivo 10 software.

**Timeline**

| **Implementation Schedule** | **2016** | | | | **2017** | | | | **2018** | | | |
| --- | --- | --- | --- | --- | --- | --- | --- | --- | --- | --- | --- | --- |
|  | **Q1** | **Q2** | **Q3** | **Q4** | **Q1** | **Q2** | **Q3** | **Q4** | **Q1** | **Q2** | **Q3** | **Q4** |
| Finalize Protocol and Interview Guide |  |  |  |  |  |  |  |  |  |  |  |  |
| Introductory Meeting and Presentation with DUKE-NUS |  |  |  |  |  |  |  |  |  |  |  |  |
| Training of Research Coordinators and Country Teams *(Session 1: Overall Approach)* |  |  |  |  |  |  |  |  |  |  |  |  |
| Training of Research Coordinators and Country Teams *(Session 2: How to Interview)* |  |  |  |  |  |  |  |  |  |  |  |  |
| Training of Research Coordinators and Country Teams *(Session 3: Data Analysis and Writing up Manuscripts)* |  |  |  |  |  |  |  |  |  |  |  |  |
| In-depth Interviews Conducted by Country Teams |  |  |  |  |  |  |  |  |  |  |  |  |
| Translation and Transcription of Interview Transcripts |  |  |  |  |  |  |  |  |  |  |  |  |
| Analysis of Qualitative Data by Country Research Teams and DUKE-NUS |  |  |  |  |  |  |  |  |  |  |  |  |
| Manuscript writing |  |  |  |  |  |  |  |  |  |  |  |  |
