# Supplementary material for: Post-intervention acceptability of multicomponent intervention for management of hypertension in rural Bangladesh, Pakistan, and Sri Lanka- a qualitative study
Source: PLoS One. 2023 Jan 19;18(1):e0280455. doi: 10.1371/journal.pone.0280455 (PMC9851540; doi:10.1371/journal.pone.0280455)
Supplement: S2 File — (PDF) [file pone.0280455.s003.pdf]

COBRA-BPS Study Group Contributors:

Main PI and country PI: Professor Tazeen H Jafar (overall principal investigator), Dr. Aliya Naheed (site principal investigator - Bangladesh), Dr. Imtiaz Jehan (site principal investigator - Pakistan), Professor Asita de Silva (site principal investigator - Sri Lanka).

Duke-NUS Medical School contributors: Professor Tazeen H Jafar (overall principal investigator, clinician-scientist/health systems trialist), Dr. Mihir Gandhi (biostatistician), Professor Eric Finkelstein (main health economist), Dr. Helena Legido-Quigley (social scientist), Dr. Marcel Bilger (health economist), Dr. Feng Liang (senior research fellow and project manager); Dr. Saeidah Tavajoh (research associate project coordinator), Mr. Lim Ching Wee (research assistant), Mr. Anirudh Krishnan (research assistant), Ms. Cecille Lintag (administrative executive).

Singapore Clinical Research Institute: Dr. Mihir Gandhi (trial statistician), Dr. Pryseley Nkouibert Assam (supporting trial statistician), Mr. Rajesh Babu Moorakonda (statistical analyst), Associate Professor Edwin Chan (epidemiologist), Mr. Zheng Yiheng (REDCap administrator).

ICDDR, B: Dr. Aliya Naheed (country PI), Prof John D Clemens (country Co-I) , Dr. Ali Tanweer, Dr. Mohammad Hasnat (country collaborator), Nantu Chakma (coordinator) , Dr. Dewan Alam, Ms. Sonia Pervin, Dr. Rubhana Raqib, Shyfuddin Ahmed, Muhammad Ashique Haider Chowdhury.

Aga Khan University: Dr. Imtiaz Jehan (country PI), Dr. Aamir Hameed (country co-I), Dr. Sahar Senan, Dr. Hamid Farazdiq, Dr. Gulshan Himani, Dr. Hunaina Shahab, Ms. Ayesha Khan (consultant).

University of Kelaniya: Professor Asita de Silva (country PI), Dr. Anurhadhani Kasuriratne (co-I), Dr. Nathasha Luke, Dr. Chamini de Silva, Dr. Manuja Perera, Dr. Channa Ranasinha, Dr. Dileepa Ediriweera.

UK-based collaborator: Professor Shah Ebrahim.

US-based collaborators: Dr. Elizabeth Turner (validation statistician).

Source of funding: The Joint Global Health Trials funding from The Department of Health and Social Care, the Department for International Development, the Global Challenges Research Fund, the Medical Research Council and Wellcome Trust, UK.

Coordinating center: Duke-NUS Medical School, Singapore.

Trial steering committee members: Professor Joep Perk (chair), Dr. Richard Smith, Professor Anne Mills, Dr. Elizabeth Allen, Professor Kate Hunt.

Funding agency observer: Ms. Jill Jones**,** the Medical Research Council, UK.

Data safety & monitoring committee: Professor Andrew Farmer (chair), Professor Doris Young, Professor Bruce Neal, Associate Professor Tan Say Beng.
