# Supplementary material for: Post-intervention acceptability of multicomponent intervention for management of hypertension in rural Bangladesh, Pakistan, and Sri Lanka- a qualitative study
Source: PLoS One. 2023 Jan 19;18(1):e0280455. doi: 10.1371/journal.pone.0280455 (PMC9851540; doi:10.1371/journal.pone.0280455)
Supplement: S3 File — (PDF) [file pone.0280455.s004.pdf]

Code list-Providers

COBRA-BPS study

Component	Codes
Acceptability	Available programs of hypertension prevention and control
	Task/responsibility of available hypertension prevention and control
	Activities during COBRA program
	Delivered HHE topics
	Providers opinion about HHE
	Participation of family members in HHE
	Patients opinion about HHE
	Opinion about BCC materials
	Experience/opinion about BP monitoring at home
	Experience of patient referral
	Opinion and suggestion about algorithm
	Opinion about GP management checklist
	Opinion about COBRA program
Implement ability	Current status of the COBRA program
	Problems faced in home visit
	Problems faced in implementing HHE
	Solution of problems in implementing HHE
	Challenges faced with BCC materials
	Recommendation for BCC materials
	Problems faced in implementing BP monitoring
	Solution of problems in implementing BP monitoring
	Problems face in patients referral
	Solution of referral problems
	Experience of medication adherence
	Reasons of non adherence of medication
	Problems of functioning NCD corner
	Solution of functioning NCD corner
Benefit	Usefulness HHE
	Usefulness BP monitoring
	Usefulness GP referral
	Usefulness of Algorithm
	Positive impact of COBRA program to the providers
	Positive impact of COBRA program to the system
Demand and Integration	Ways to support for poor and dependent patients
	Recommendation of COBRA program
	Opinion about COBRA scale up
	Suggestions for scaling up of the COBRA
