# Supplementary material for: Post-intervention acceptability of multicomponent intervention for management of hypertension in rural Bangladesh, Pakistan, and Sri Lanka- a qualitative study
Source: PLoS One. 2023 Jan 19;18(1):e0280455. doi: 10.1371/journal.pone.0280455 (PMC9851540; doi:10.1371/journal.pone.0280455)
Supplement: S4 File — (PDF) [file pone.0280455.s005.pdf]

Code list-Patients part

Component	Codes (30)
Acceptability	Acc-Health assistant visit
	Health assistant activities
	Acc-Participation in HHE
	Acc-Opinion about HHE
	Acc-Opinion about BP monitoring
	Acc-Referral for hypertension treatment
	Acc-Utilization of referral
	Acc-Experience of UHC visit
	Acc-Collection of medicine
Component	Codes
Benefit	Ben-Usefulness of HHE and BP monitoring
	Ben-Dietary modifications
	Ben-Change in physical activity
	Ben-Change in tobacco use
	Ben-Medication adherence
	Ben-Stress reduction
	Ben-Differences observed
Component	Codes
Implement ability	Imp-Problems of HHE
	Imp-Problems of BP monitoring
	Imp-Medicine supply from UHC
	Bar-Reason for not receiving medicine from UHC
	Bar-Reason for non adherence of medication
	Bar-Reasons for not visiting UHC
Component	Codes
Scalability	Sca-Prospects of COBRA intervention
Component	Codes
Demand	Dem-Areas need to be improved for HHE
	Dem-Areas need to be improved for BP monitoring
	Dem-Recommendations for improving HHE
	Dem-Recommendations for improving BP monitoring
	Dem-Recommendations for improving hypertension service at UHC
	Dem-Recommendations of poor and dependent population
