# Supplementary material for: Post-intervention acceptability of multicomponent intervention for management of hypertension in rural Bangladesh, Pakistan, and Sri Lanka- a qualitative study
Source: PLoS One. 2023 Jan 19;18(1):e0280455. doi: 10.1371/journal.pone.0280455 (PMC9851540; doi:10.1371/journal.pone.0280455)
Supplement: S5 File — (PDF) [file pone.0280455.s006.pdf]

Healthcare Providers Interview Probes

a) **Perceived usefulness of care strategy**

How has the training **changed the way you manage patients** for hypertension? (e.g. measuring BP/providing lifestyle advice/prescribing therapy/increasing adherence/referring patients)

How have **patient outcomes** (e.g. control of blood pressure) or **patient behavior** (e.g. lifestyle factors) related to hypertension **changed**, since you implemented strategies learnt in the training?

Overall, how **satisfied** are you with the training you have received in managing hypertension in patients?

b) **Impact of incorporating care strategy on existing practice**

How has implementing these care strategies on hypertension **changed your day-to-day practice at work**?

- How has your caseload changed?
- How has the amount of time spent with each patient changed?
- How has interaction with other healthcare providers changed?
- **For MLPs only:** were you able to reach a physician when you wanted to ask a question while treating hypertension?
- **For MLPs only:** were physicians available to sign/counter-sign your prescriptions for antihypertensives? Please elaborate on these details in your practise setting.
- **For physicians only:** If your practice has MLPs, Were you able to adequately supervise MLPs?
- **For physicians only:** If your practice has MLPs, were they able to ask you questions when they needed to? Were you available to sign/counter-sign prescriptions written by MLPs for antihypertensives? Please elaborate on these details in your practice setting

c) **Barriers and facilitators to integrating care strategy**

What were some **difficulties you faced** when you incorporated these changes in hypertension management in your practice?

What could have **helped you more successfully incorporate** these changes in hypertension management in your practice?

d) **Attitude toward participation in full-scale implementation**

What is your **overall opinion** about this program?

If we were to conduct annual training for the program, **would you return**?

If yes, what would be your **main concerns?** Which **areas are most important to address?** (e.g. training and non-training related areas)

COMMUNITY HEALTH WORKERS SPECIFIC INTERVIEW PROBES

a) **Perceived usefulness of care strategy**

How well did household participants **engage** in the home health education provided on hypertension?

- Were children included in the sessions?
- Were males included in the sessions?

b) Impact of incorporating care strategy on existing practice

How has providing home health education on hypertension **changed your day-to-day practice at work**?

- How has your caseload changed?
- How has the amount of time spent with each household changed?
- How has the delivery of your other duties changed?

c) Barriers and facilitators to integrating care strategy

- What were some **difficulties you faced** when you incorporated home health education of hypertension into your current duties?
- What could have **helped you more successfully incorporate** home health education of hypertension into your current duties? (e.g. incentives)

d) Attitude toward participation in full-scale RCT and implementation

- What is your **overall opinion** about this program?
- If we were to incorporate home health education on hypertension in the duties of community health workers, what would be your **main concerns?** Which **areas are most important to address?**
