# Supplementary material for: Post-intervention acceptability of multicomponent intervention for management of hypertension in rural Bangladesh, Pakistan, and Sri Lanka- a qualitative study
Source: PLoS One. 2023 Jan 19;18(1):e0280455. doi: 10.1371/journal.pone.0280455 (PMC9851540; doi:10.1371/journal.pone.0280455)
Supplement: S6 File — (PDF) [file pone.0280455.s007.pdf]

PATIENT INTERVIEW PROBES

a) Experience during CHW visit

Could you tell me, when the CHW visited you in the past 2 to 3 months, what did they talk to you about? For example:

- Obesity
- High blood pressure
- Physical activity

If you were referred to a GP, were you **satisfied** with the **referral process**?

b) Comparing usual care to CHW visit

Was this visit by the CHW **any different** from your usual visits by CHWs?

- If yes, how was it different?
- What additional services did the CHW provide?
- Were the topics discussed different? Did the CHW tell you something new about blood pressure that you didn't already know?
- Did you like what was different during the visit?
- If your BP was elevated, were you referred to the GP?
- Were you given a referral checklist to visit the GP? Did you bring it with you to the GP?

c) Perceived usefulness of CHW visit

Was the information shared with you regarding your high blood pressure by the CHW useful to you?

- Were you able to understand what the CHW was telling you?
  - If no, what was unclear?
- Are you able to do the things that your CHW has advised you to do? (e.g. lifestyle changes, taking your medications)

Did you **feel** the referral process from CHW to GP was **useful?**

d) Experience during intervention visit to GP

Could you tell me, when you visited your healthcare provider (MLP or PP), what did your doctor/provider discuss with you about your blood pressure during your clinic visit? For example:

- Current blood pressure level
- Explaining disease condition
- Medication, e.g. how to take your medication, what side effects are experienced, what side effects are expected
- Lifestyle advice

e) Perceived usefulness of intervention visits to GP

Was the **information shared** with you regarding your high blood pressure **useful** to you?

- Were you **able to understand** what your doctor/provider was telling you?
- **Are you able to do** the things that your doctor/provider has advised you to do? (e.g. lifestyle changes, taking your medications)
  - Why was it difficult to follow?

- After that session, do you **feel** that you **understand your condition better**, including how to manage this condition?
    - If no, what do you need to know about the condition?

f) Overall attitude and confidence about managing one's blood pressure

Overall, do you **feel you understand** your blood pressure condition?

- If not, what else would you need to know?

Do you **feel confident** about **managing your blood pressure**?

- If not, what else would you need?
